# Supplementary material for: Resolving the full spectrum of human genome variation using Linked-Reads
Source: Genome Res. 2019 Apr;29(4):635–45. doi: 10.1101/gr.234443.118 (PMC6442396; doi:10.1101/gr.234443.118)
Supplement: Supplemental Material [file supp_gr.234443.118_Supplemental_longranger-2.2.zip › longranger-2.2.tar.bz2!/lr2.2-public/lib/stan/cmdstan/stan/doc/api/html/covar__adaptation_8hpp_source.html]

Stan: src/stan/mcmc/covar\_adaptation.hpp Source File


|  |  |
| --- | --- |
|  | Stan  2.5.0  probability, sampling & optimization |


- Main Page
- Related Pages
- Namespaces
- Classes
- Files

- File List
- File Members

All Classes Namespaces Files Functions Variables Typedefs Enumerations Enumerator Friends Macros Pages

- src
- stan
- mcmc

covar\_adaptation.hpp

Go to the documentation of this file.

1 #ifndef STAN\_\_MCMC\_\_COVAR\_\_ADAPTATION\_\_BETA

2 #define STAN\_\_MCMC\_\_COVAR\_\_ADAPTATION\_\_BETA

3

4 #include <vector>

5 #include <stan/math/matrix/Eigen.hpp>

6

7 #include <stan/prob/welford\_covar\_estimator.hpp>

8 #include <stan/mcmc/windowed\_adaptation.hpp>

9

10 namespace stan {

11

12  namespace mcmc {

13

14  class covar\_adaptation: public windowed\_adaptation {

15

16  public:

17

18  covar\_adaptation(int n): windowed\_adaptation("covariance"), estimator\_(n) {}

19

20  bool learn\_covariance(Eigen::MatrixXd& covar, const Eigen::VectorXd& q) {

21

22  if (adaptation\_window()) estimator\_.add\_sample(q);

23

24  if (end\_adaptation\_window()) {

25

26  compute\_next\_window();

27

28  estimator\_.sample\_covariance(covar);

29

30  double n = static\_cast<double>(estimator\_.num\_samples());

31  covar = (n / (n + 5.0)) \* covar

32  + 1e-3 \* (5.0 / (n + 5.0)) \* Eigen::MatrixXd::Identity(covar.rows(), covar.cols());

33

34  estimator\_.restart();

35

36  ++adapt\_window\_counter\_;

37  return true;

38

39  }

40

41  ++adapt\_window\_counter\_;

42  return false;

43

44  }

45

46  protected:

47

48  prob::welford\_covar\_estimator estimator\_;

49

50  };

51

52  } // mcmc

53

54 } // stan

55

56 #endif

stan::prob::welford\_covar\_estimator::num\_samples

int num\_samples()

**Definition:** welford\_covar\_estimator.hpp:35

windowed\_adaptation.hpp

stan::prob::welford\_covar\_estimator::sample\_covariance

void sample\_covariance(Eigen::MatrixXd &covar)

**Definition:** welford\_covar\_estimator.hpp:39

stan::mcmc::windowed\_adaptation::end\_adaptation\_window

bool end\_adaptation\_window()

**Definition:** windowed\_adaptation.hpp:83

stan::mcmc::windowed\_adaptation

**Definition:** windowed\_adaptation.hpp:13

stan::mcmc::windowed\_adaptation::adaptation\_window

bool adaptation\_window()

**Definition:** windowed\_adaptation.hpp:77

stan::mcmc::covar\_adaptation

**Definition:** covar\_adaptation.hpp:14

stan::mcmc::covar\_adaptation::estimator\_

prob::welford\_covar\_estimator estimator\_

**Definition:** covar\_adaptation.hpp:48

stan::mcmc::covar\_adaptation::learn\_covariance

bool learn\_covariance(Eigen::MatrixXd &covar, const Eigen::VectorXd &q)

**Definition:** covar\_adaptation.hpp:20

Eigen.hpp

stan::mcmc::windowed\_adaptation::compute\_next\_window

void compute\_next\_window()

**Definition:** windowed\_adaptation.hpp:88

stan::math::e

double e()

Return the base of the natural logarithm.

**Definition:** constants.hpp:86

welford\_covar\_estimator.hpp

stan::mcmc::covar\_adaptation::covar\_adaptation

covar\_adaptation(int n)

**Definition:** covar\_adaptation.hpp:18

stan::prob::welford\_covar\_estimator

**Definition:** welford\_covar\_estimator.hpp:11

stan::mcmc::windowed\_adaptation::adapt\_window\_counter\_

unsigned int adapt\_window\_counter\_

**Definition:** windowed\_adaptation.hpp:117

stan::prob::welford\_covar\_estimator::restart

void restart()

**Definition:** welford\_covar\_estimator.hpp:19

stan::prob::welford\_covar\_estimator::add\_sample

void add\_sample(const Eigen::VectorXd &q)

**Definition:** welford\_covar\_estimator.hpp:25


---

[ Stan Home Page ]

*© 2011–2014,
Stan Development Team.*
